# 

**Published:** 1860-01

**Authors:** 


### LIST OF COLLABORATORS.

ARMOR, S. G., M.D.,
Late Professor of Pathology and Clinical Medicine, Missouri Medical College.

ATLEE, JOHN L., M.D.,
Late President Pennsylvania State Medical Society.

ATLEE, WALTER F., M.D.,
Philadelphia.

BARCLAY, ROBERT G., M.D.,
Jerusalem, Syria.

BELL, JOHN, M D.,
Philadelphia, formerly Professor of Medicine, Medical College of Ohio.

BEMISS, S. M., M.D.,
Professor of Clinical Medicine, University of Louisville.

BLACKBURN, L. P., M.D.,
Natchez, Miss.

BLACKIE, GEORGE C., M.D.,
Professor of Botany, University of Nashville.

BLACKMAN, G. C., M.D.,
Professor of Surgery, Medical College of Ohio.

BOBBS, J. L., M.D.,
Formerly Professor of Anatomy, Indianapolis.

BOWEN, W. S., M.D.,
New York.

BOZEMAN, N., M.D.,
Montgomery, Ala.

BRADFORD, J. T., M.D.,
Augusta, Ky.

BRINTON, JOHN H., M.D.,
Lecturer on Operative Surgery, Philadelphia.

CARNOCHAN, J. M., M.D.,
Professor of Surgery in the New York Medical College.

CHIPLEY, W. S., M.D.,
Professor of Medicine, Transylvania University.

CLAPP, A., M.D.,
New Albany, Indiana.

CLIFFE, D. B., M.D.,
Franklin, Tenn.

DA COSTA, J., M.D.,
Lecturer on the Practice of Medicine at the Philadelphia Medical Association.

DICKSON, SAMUEL H., M.D.,
Professor of Medicine, Jefferson Medical College.

DUNGLISON, RICHARD J., M.D.,
Philadelphia.

DUPIERRIS, M., M.D.,
Havana, Cuba.

EDGAR, W. F., M.D.,
United States Army.

FARRAR, S. C., M.D.,
Jackson, Miss.

FENNER, C. S., M.D.,
Memphis, Tenn.

FISCHER, EMIL, M.D.,
Philadelphia.

FLINT, AUSTIN, M.D.,
Professor of Clinical Medicine, Auscultation, and Percussion, New Orleans School of Medicine.

FRICK, CHARLES, M.D.,
Professor of Materia Medica, University of Maryland.

GADBURY, W. Y., M.D.,
Benton, Miss.

GIBBS, O. C., M.D.,

Chautauque County, New York.

HALL, J. P., M.D.,
Glasgow, Ky.

HAMMOND, WM. A., M.D.,
United States Army.

HARDIN, JOHN, M.D.,
Professor of Obstetrics, Kentucky School of Medicine, Louisville.

HARTSHORNE, EDWARD, M.D.,
Surgeon to the Pennsylvania Hospital.

HASKINS, E. B., M.D.,
Professor of Medicine, Shelby Medical College, Nashville, Tenn.

HENTZ, C. A., M.D.,
Marianna, Florida.

HEWSON, ADDINELL, M.D.,
One of the Surgeons to Wills Hospital for Diseases of the Eye, Philadelphia.

HOLLINGSWORTH, SAMUEL L., M.D.,
Formerly Editor of the Medical Examiner, Philadelphia.

JEWELL, WILSON, M.D.,
Philadelphia, formerly President of the Quarantine and Sanitary Convention.

KEATING, WM. V., M.D.,
Lecturer on Obstetrics and the Diseases of Women, at the Philadelphia Medical Association.

KERR, JOHN G., M.D.,
Canton, China.

KUNKLER, G. A., M.D.,
Madison, Ia.

LOGAN, BEN., M.D.,
Shreveport, La.

LOPEZ, A., M.D.,
Mobile, Ala., formerly President of the Alabama State Medical Society.

MEIGS, J. AITKEN, M.D.,
Professor of the Institutes of Medicine in the Medical Department of Pennsylvania College.

MITCHELL, S. WEIR, M.D.,
Lecturer on Physiology at the Philadelphia Medical Association.

MOREHOUSE, GEORGE, R., M.D.,
Philadelphia.

RAPHAEL, B. I., M.D.,
New York.

REEVE, J. C., M.D.,
Dayton, Ohio,

RIVES, L. C., M.D.,
Formerly Professor of Midwifery, Medical College of Ohio.

SMITH, J. LAWRENCE, M.D.,
Professor of Chemistry, University of Louisville.

SNEED, W. C., M.D.,
Frankfort, Ky., formerly President of Kentucky State Medical Society.

SPILMAN, C. H., M.D.,
Harrodsburg, Ky., formerly President of Kentucky State Medical Society.

STORER, HORATIO R., M.D.,
Formerly one of the Physicians to the Boston Lying-in Hospital, Boston, Mass.

SUTTON, GEO., M.D.,
Aurora, Ia.

SUTTON, W. L., M.D.,
Georgetown, Ky., formerly President Kentucky State Medical Society.

THOMPSON, S., M.D.,
Albion, Ill., formerly President of the Illinois State Medical Society.

TODD, L. BEECHER, M.D.,
Lexington, Kentucky.

WILLIAMS, E., M.D.,

Cincinnati, Ohio.

### Bi-Monthly Bibliographical Record.

AMERICAN.

Caloric: its Mechanical, Chemical, and Vital Agencies in the Phenomena of Nature. By the late Samuel L. Metcalfe, M.D. New edition. 8vo., pp. 640 and 480. Philadelphia: J. B. Lippincott & Co., 1859. (From the publishers.)

Proceedings and Debates of the Third National Quarantine and Sanitary Convention, held in the City of New York, April, 1859. 8vo., pp. 728. New York, 1859. (From Dr. Francis.)

Transactions of the American Medical Association. Vol. xii. 8vo., pp. 722. Philadelphia, 1859. (From the Secretary.)

Transactions of the Indiana State Medical Society, at its Tenth Annual Session, held in the City of Indianapolis, May 17th, 1859. Pamphlet, 8vo., pp. 48. Indianapolis, 1859. (From the Secretary.)

Ninth Annual Meeting of the Illinois State Medical Society, held in Decatur, June, 1859. Pamphlet, 8vo., pp. 146. Chicago, 1859. (From the Secretary.)

The Transactions of the New York Academy of Medicine; containing Remarks upon Chylous, or Milky Urine. By C. E. Isaacs, M.D. Part iv., vol. ii. Pamphlet, 8vo., pp. 77–96. New York, 1859. (From Dr. Isaacs.)

The Life and Labors of Laennec: an Introductory Address, delivered at the New Orleans School of Medicine, Nov. 14th, 1859. By Austin Flint, M.D. Reprint, 8vo., pp. 20. New Orleans, 1859. (From the author.)

Description of a Deformed, Fragmentary Human Skull, etc. By J. Aitken Meigs, M.D. Pamphlet, 8vo., pp. 19. Philadelphia, 1859. (From the author.)

An Introductory Lecture, delivered in the Philadelphia School of Anatomy, Oct. 17th, 1859. By Dr. Hayes Agnew, M.D. Pamphlet, 8vo., pp. 16. Philadelphia, 1859. (From the author.)

Introductory Lecture to the Course on Midwifery and Diseases of Women and Children, in the Jefferson Medical College, delivered Oct. 12th, 1859. By Charles D. Meigs, M.D. Pamphlet, 8vo., pp. 21. Philadelphia, 1859. (From the author.)

Method of Education; an Address Introductory to the Session 1859–60 of the St. Louis Medical College. By T. H. Watters, M.D. Pamphlet, 8vo., pp. 20. St. Louis, 1859. (From the author.)

The Medical Profession and its Claims; being an Introductory Lecture to the Winter Session of the New York Medical College of 1859–60. By James Bryan, M.D. Pamphlet, 8vo., pp. 25. New York, 1859. (From the author.)

Botany as an Ally of Medicine; a Lecture delivered to the Medical Society of the University of Nashville, Dec. 2d, 1859. By George S. Blackie, M.D. Pamphlet, 8vo., pp. 24. Nashville, 1859. (From the author.)

Proceedings of the Academy of Natural Sciences of Philadelphia. 8vo., pp. 287–326. Philadelphia, 1859. (From the Secretary.)

Records of Daily Practice; a Scientific Visiting List for Physicians and Surgeons. By A. N. Bell, M.D. 12mo. New York: Baillière & Brothers, 1859. (From the publishers.)

FOREIGN.

Phthisis and the Stethoscope; or, the Physical Signs of Consumption. By Richard Payne Cotton, M.D. Foolscap 8vo. Second edition. London: Churchill, 1859.

Illustrations of a Peculiar Bleeding Tumor in the Rectum. By Richard Quain, F.R.S. Small 8vo. London: Walton & Maberly, 1859.

Traité Complet de Chimie Analytique. By Henri Rose. Tome i., (Analyse Qualificative,) 2d fasic. pp. 481–1063. Paris: Bossange et Fils, 1859.

A Practical Treatise on Diseases of the Heart, Lungs, and Air-Passages; with a Review of the several Climates recommended in these Affections. By James Bright, M.D. Third edition. Post 8vo. London: Churchill, 1859.

Lectures on the Development of the Gravid Uterus. By Wm. O. Priestly, M.D. 8vo. London: Churchill, 1859.

On the Injurious Effects of Mercury in the Treatment of Disease. By S. O. Habershon, M.D. Post 8vo. London: Churchill, 1859.

Traité Pratique de l'Hygiène et des Maladies de l'Enfance. By E. Le Barillier, M.D. 12mo. Paris: Bossange et Fils, 1859.

Traité d'Hétérogénie, ou de la Génération Spontanée, basé sur l'Expérimentation. By F. A. Pouchet. 8vo. Paris: Bossange et Fils, 1859.

Blood Disease. By T. Vaughan Hughes, M.D. Crown 8vo., pp. 132. London: Churchill, 1859.

